# Identification, Functional Characterization, and Regulon Prediction of the Zinc Uptake Regulator (*zur*) of *Bacillus anthracis* – An Insight Into the Zinc Homeostasis of the Pathogen

**DOI:** 10.3389/fmicb.2018.03314

**Published:** 2019-01-11

**Authors:** Divya Kandari, Monisha Gopalani, Manish Gupta, Hemant Joshi, Sonika Bhatnagar, Rakesh Bhatnagar

**Affiliations:** ^1^Laboratory of Molecular Biology and Genetic Engineering, School of Biotechnology, Jawaharlal Nehru University, New Delhi, India; ^2^Computational and Structural Biology Laboratory, Division of Biotechnology, Netaji Subhas Institute of Technology, University of Delhi, New Delhi, India

**Keywords:** *Bacillus anthracis*, zinc homeostasis, regulon, zinc uptake system, zinc mobilization, metal chaperones, autoregulation

## Abstract

Zinc has an abounding occurrence in the prokaryotes and plays paramount roles including catalytic, structural, and regulatory. Zinc uptake regulator (Zur), a Fur family transcriptional regulator, is connoted in maintaining zinc homeostasis in the pathogenic bacteria by binding to zinc and regulating the genes involved in zinc uptake and mobilization. Zinc homeostasis has been marginally scrutinized in *Bacillus anthracis*, the top-rated bio-terror agent, with no decipherment of the role of Zur. Of the three Fur family regulators in *B. anthracis*, BAS4181 is annotated as a zinc-specific transcriptional regulator. This annotation was further substantiated by our stringent computational and experimental analyses. The residues critical for zinc and DNA binding were delineated by homology modeling and sequence/structure analysis. *ba zur* existed as a part of a three-gene operon. Purified BaZur prodigiously existed in the dimeric form, indicated by size exclusion chromatography and blue native-polyacrylamide gel electrophoresis (PAGE). Computational and manual strategies were employed to decipher the putative regulon of *ba zur*, comprising of 11 genes, controlled by six promoters, each harboring at least one Zur box. The DNA binding capability of the purified BaZur to the upstream regions of the *ba zur* operon, *yciC*, *rpmG*, *znuA*, and genes encoding a GTPase cobalamine synthesis protein and a permease was ascertained by electrophoretic mobility shift assays. The regulon genes, implicated in zinc uptake and mobilization, were mostly negatively regulated by BaZur. The *ba zur* expression was downregulated upon exposure of cells to an excess of zinc. Conversely, it exhibited a marked upregulation under N, N, N′, N′-Tetrakis (2-pyridylmethyl) ethylenediamine (TPEN) mediated zinc-depleted environment, adding credence to its negative autoregulation. Moreover, an increase in the transcript levels of the regulon genes *znuA*, *rpmG*, and *yciC* upon exposure of cells to TPEN connoted their role in combating hypo-zincemic conditions by bringing about zinc uptake and mobilization. Thus, this study functionally characterizes Zur of *B. anthracis* and elucidates its role in maintaining zinc homeostasis.

## Introduction

Zinc has the stature of being the second most plenteously occurring transition element in the living systems after iron. The gamut of functions that it plays, including catalytic, structural, and regulatory, both in bacteria and higher organisms accentuates its significance. Approximately 5–6% of the bacterial proteins exhibit zinc-dependency (Hantke, 2005; Andreini et al., 2006; Rahman and Karim, 2018). The metal acts as an electrophile or Lewis acid in most of the hydrolytic reactions, thereby catalyzing them and is also incorporated into a variety of metalloenzymes, storage proteins, and transcription factors (Falchuk, 1993; Coleman, 1998; Auld, 2001; Outten and O’halloran, 2001; Cerasi et al., 2013). Next, it can act both as an antioxidant, protecting the sulfhydryl groups of proteins from the attack by the free radicals, and as a combatant preventing the formation of free radicals, thus, perpetuating the structural stability and functionality of several proteins (Bray and Bettger, 1990). However, zinc can be toxic to the cells at a concentration higher than the normal by either blocking the thiols of the proteins or by competitive mismetallation with the binding sites of the other metal ions in them, consequently precluding various physiological processes (Beard et al., 1995; Mills et al., 2002; Imlay, 2014; Chandrangsu et al., 2017).

Upon pathogen invasion, one of the intrinsic defensive strategies that the host brings into play is the nutritional immunity, wherein the host either abstains or intoxicates the pathogen with the essential transition elements; zinc being one of them, thus abating its survival. Therefore, successful infection and replication require the pathogen to adapt to the hypo-zincemic or hyper-zincemic conditions within the host (Cassat and Skaar, 2012; Hood and Skaar, 2012; Vignesh and Deepe, 2016). Furthermore, there are many zinc-dependent virulence factors that contribute to the survival and pathogenesis of the bacteria. Broadly, the genes implicated in metal homeostasis in the pathogenic bacteria have been manifested as virulence determinants (Botella et al., 2011; Cheryl-lynn et al., 2015; Djoko et al., 2015).

Owing to the aforementioned crucial roles that zinc plays in the bacteria, maintenance of extracellular and intracellular zinc homeostasis is imperative at all times. For this the bacteria, precisely the pathogens have evolved intricate strategies like regulation at the transcriptional level brought about by the metal sensing regulators, zinc purging, and acquisition from the environment, and increasing the expression of non-zinc requiring proteins, thereby allocating zinc to the zinc-dependent proteins (Hantke, 2001, 2005; Rudolph et al., 2006; Fillat, 2014).

*Bacillus anthracis*, a Gram-positive, spore-forming bacterium is the causative agent of the fatal disease anthrax. It is essentially a zoonotic disease; however, humans occasionally acquire the infection upon close proximity to the infected animals or the contaminated animal products (Mock and Fouet, 2001). The fact that the spores of this pathogen can remain viable for decades and can be subjected to weaponization and dissemination as aerosols with ease, augments its potential to be used as bio-terror or bio-warfare agent. The same was exemplified by the spores attack via the US postal mail service in the year 2001 (Hudson et al., 2008). The key virulence determinants of the pathogen are plasmid encoded including a poly-γ-d-glutamic acid capsule and a tripartite toxin that comprises of three structural components, namely, protective antigen (PA), lethal factor (LF), and edema factor (EF). The binary combination of PA with LF and EF results in the formation of lethal (LT) and edema toxins (ET), respectively. Since LF is a Zn^+2^-dependent metalloprotease, in addition to the physiologically cardinal roles that zinc plays, it is also essential for the activity of the LT of *B. anthracis* (Klimpel et al., 1994; Duesbery et al., 1998; Bradley et al., 2001; Mock and Fouet, 2001; Scobie et al., 2003; Guichard et al., 2012). Thusly, maintaining zinc homeostasis becomes even more pivotal in the pathogen. However, zinc homeostasis in *B. anthracis* remains majorly unexplored and thus needs an insightful scrutinization.

Zinc uptake regulator (Zur) belongs to the FUR superfamily, comprising of the metal sensing transcriptional regulators, which bring about transition metal homeostasis in the bacteria by pertinent alterations in its gene expression. The members of this superfamily are pervasive among the prokaryotes and in addition to Zur it includes Fur (ferric uptake regulator) (Escolar et al., 1999; Hantke, 2002), Mur (manganese uptake regulator) (Diaz-Mireles et al., 2004; Platero et al., 2007), Nur (nickel uptake regulator) (Ahn et al., 2006), and Per (peroxide regulator) (Verneuil et al., 2005; Lee and Helmann, 2006) proteins (Fillat, 2014). Zur, a paramountly significant protein in zinc homeostasis, was first discovered in *Escherichia coli* (Patzer and Hantke, 1998) and has now been extensively reviewed in a variety of organisms including *Bacillus subtilis* (Gaballa and Helmann, 1998; Prestel et al., 2015; Shin and Helmann, 2016), *Streptococcus sp* (Feng et al., 2008), *Listeria monocytogenes* (Dalet et al., 1999), *Staphylococcus aureus* (Lindsay and Foster, 2001), *Salmonella enterica* (Campoy et al., 2002), *Yersinia pestis* (Li et al., 2009), *Streptomyces coelicolor* (Shin et al., 2007), *Enterococcus faecalis* (Latorre et al., 2015), *Xanthomonas campestris* (Tang et al., 2005)*, Mycobacterium tuberculosis* (Maciąg et al., 2007), and *Pseudomonas aeruginosa* (Ellison et al., 2013). In bacteria, Zur orchestrates the fluctuations in the intracellular and extracellular zinc levels with appropriate changes in the transcriptome, by regulating the expression of the genes involved in zinc homeostasis. Under zinc sufficiency, Zur exists in a zinc bound form, capable of binding to DNA and keeping the genes involved in zinc uptake and mobilization in a repressed state. However, this Zur mediated repression is released when the cell encounters a zinc depleted condition, thereby initiating zinc uptake and mobilization (Patzer and Hantke, 1998). Most often, the regulon of Zur comprises of genes that encode for low- and high-affinity zinc transporters, chaperonic proteins, the non-zinc binding paralogs of the ribosomal proteins, and other proteins involved in zinc homeostasis (Fillat, 2014). Moreover, Zur has also been documented to play role in the pathogenesis of *S. enterica* (Campoy et al., 2002), *X. campestris* (Tang et al., 2005; Yang et al., 2007), and *L. monocytogenes* (Dowd et al., 2012).

The present study aims at the identification and functional characterization of the zinc uptake regulator of *B*. *anthracis*, a transcriptional factor that is indispensable for maintaining zinc homeostasis. Of the three Fur family proteins co-existing in *B. anthracis*, the BAS4181 locus was annotated as *ba zur* with high precision by using a comprehensive *in silico* sequence analysis, followed by homology modeling. We report the genomic organization of the operon and biochemical properties of the BaZur protein including its dimeric propensity and DNA binding capability. Most of the regulon genes were implicated in zinc uptake, mobilization, and as metal chaperones and were negatively regulated by BaZur. Transcriptional regulation of *ba zur* was ascertained in different growth phases of *B. anthracis*, under conditions of zinc excess, and TPEN mediated zinc deprivation. Further, transcript levels of the regulon genes were also analyzed under zinc deprived conditions.

## Materials and Methods

### Bacterial Strain, Plasmid, and Culture Conditions

*Escherichia coli* DH5α and BL21 (λDE3) strains were used as cloning and expression hosts, respectively. The strains were grown in Luria-Bertani (LB) medium, supplemented with antibiotics, ampicillin (100 μg/mL) and kanamycin (50 μg/mL) as required. An avirulent *B. anthracis* Sterne 34F2 strain (pXO1^+^ pXO2^−^) was used in this study and cultured in brain heart infusion (BHI), LB, or minimal medium (M9) as per the requirement of the experiment. For ascertaining the growth-specific difference in the expression of *ba zur* by qRT-PCR, *B. anthracis* cells were grown in BHI medium at 37°C up to an O.D._600 nm_ of ∼0.3, 0.6, 0.9, and 1.2, corresponding to the early-exponential, mid-exponential, late-exponential, and onset of stationary phase, respectively, and harvested. For growing *B. anthracis* cells under conditions of zinc excess, a secondary culture was set up in a 100 mL LB broth flask using the O/N grown primary culture, followed by incubation at 37°C until the early-exponential phase (O.D._600 nm_∼0.3) was attained. At this point, the culture was divided such that one part represented the control (no exogenous zinc) and the other parts were supplemented with an excess of zinc in the form of a gradient of ZnCl_2_ from 250 μM to 2 mM. All the samples were harvested when the O.D._600 nm_ of the control, i.e., cells grown without exogenous zinc reached 0.6 (approximately 45–60 min post-division and zinc addition). Next, for inducing the zinc deprived conditions, the secondary culture of *B. anthracis* cells after attaining the early-exponential phase (O.D._600 nm_∼0.3) was divided into control (no TPEN) and the other part was supplemented with 50 μM TPEN (MP Biomedicals). The samples were then harvested 40 min post-TPEN addition.

### Sequence and Structure Analysis of BaZur

BAS4181 annotated as the zinc-specific transcriptional regulator was identified on the *B. anthracis* genome using NCBI and KEGG databases (Kanehisa et al., 2017). The operonic organization of BAS4181 (*ba zur*) was predicted using DOOR (Mao et al., 2013) and ProOpDB (Taboada et al., 2011) databases. The domain architecture of the protein was determined by CDD (NCBI) (Marchler-Bauer et al., 2014) and SMART (Letunic et al., 2014) databases. Next, the *ba zur* locus was compared with that of the other bacteria. The conserved residues in BaZur were delineated by identifying the homologs of the protein in other species by BLASTP, followed by their alignment using the ClustalW multiple sequence alignment server (McWilliam et al., 2013).

A suitable template for the homology modeling of BaZur was identified by BLASTP search at PDB, using the 137 aa BaZur (NCBI protein ID: YP_030430) as the query sequence. The three-dimensional structure was modeled using the I-TASSER server (Yang and Zhang, 2015) with the available crystal structure of Zur from *S. coelicolor* (ScoZur) (PDB ID: 3MWM) as the template (Shin et al., 2011). The two zinc binding sites important for activity were also modeled by I-TASSER and annotated on the basis of similarity to BsuZur (Ma et al., 2011). In order to obtain the best distances for zinc and its coordinating residues, constrained minimization of the model was carried using AMBER 7 FF02 atom types and force field (Case et al., 2002). Zinc atoms were assigned a charge of +2 in SYBYL7.1 version (Tripos, Inc., St. Louis, MO, Unite States).

DNA binding proteins harboring an HTH motif usually bind to DNA by inserting their recognition helix into the major groove of the consensus sequence. There is a close structural similarity between multiple HTH-DNA complexes as indicated by a low root-mean-square deviation (RMSD < 3.5 Å) of superposition (AlQuraishi et al., 2015). This became our basis of modeling the BaZur–DNA complex. The DNA was modeled on the basis of the crystal structure of the IdeR-DNA complex of *M. tuberculosis* since IdeR consists of a helix–turn–helix (HTH) motif just like BaZur and the DNA binding sequence of IdeR consists of a 9-1-9 motif (Wisedchaisri et al., 2004), the same as that for BaZur. The nine base pair double-stranded DNA recognition motif from 1U8R (RCSB PDB ID of IdeR) was mutated into that of the BaZur using the biopolymer mutate option available at SYBYL. 7.1 (Tripos, Inc., St. Louis, MO, United States). An initial model of the complex with BaZur was obtained by superposition of the 13-residue recognition helix from BaZur (F53-E65) with that of the IdeR (G38-R50). This was used to obtain the DNA bases of the major groove in contact with the recognition helix of BaZur as A3, G4, C5, A6, G9 and C29, C30, C31. The expert interface of the HADDOCK2.2 web server (Van Zundert et al., 2016) was used for the protein-DNA docking in order to define the contacting residues as active residues and the surrounding residues as the passive residues for both BaZur and DNA. The side chains of the residues of the recognition helix were treated as flexible. The first and best structure from the largest and top-ranked cluster was taken as the model of the BaZur–DNA complex.

### Reverse Transcription PCR

The *in silico* predicted operonic organization of *ba zur* was corroborated *in vitro* by detecting the polycistronic transcripts using reverse transcription PCR (RT-PCR). For this, *B. anthracis* cells were grown up to the exponential phase (O.D._600 nm_ ∼ 0.6), followed by total RNA extraction using TRI reagent (Sigma-Aldrich, United States). The genomic DNA contamination, if any, was removed by subjecting the total RNA to DNase I treatment (RNase free DNase set, QIAGEN) and confirming the same by PCR using *ba zur* operon-specific primers (Table 1). Next, the total RNA was used as a template for the cDNA synthesis by the High-Capacity cDNA Reverse Transcription kit (Life Technologies). The RT-PCR reaction was performed using the cDNA pool as the template (25 ng) with 15 pmoles of the indicated primers (Table 1) in a 25 μL reaction mixture. The reaction products were analyzed by electrophoresis on a 1% agarose gel, followed by visualization using ethidium bromide staining.

**Table 1:** Primers used in the study.

S.No	Study/gene	Forward primer 5′–3′	Reverse primer 5′–3′
1	Cloning in pET28a+/BAS4181	AGCACCATGGCGATGAATCTAACAG	ATGCCTCGAGCTTTGCACATTTTG
2	Cloning in pET23a/BAS4181	AGCCGGATCCATGAATCTAACAGAAG	GCGCAAGCTTCTTTGCACATTTTG
3	qRT-PCR/*ba zur*	CGTTATTTAACGGCGAAAGAC	AACACCGATTTCAGCAAACAC
4	qRT-PCR/BAS1889	TTTCCCCTTGCAGATTTTGC	CAGGTGGGTAAATCGCTTCAA
5	qRT-PCR/*yciC*	AAGGAGCCGGTGAATGGATA	GGCTCTTCTATGATCATTTGATTTCTT
6	qRT-PCR/*rpmG*	GAGTGCGGTGATCGTAATTATATTCTAA	CGCTTTAATCGTGGACAATATTTTT
7	EMSA/BAS1632	GAAATTATTTCTCTCCTTTAAATCG	CCTTCTCGCCTCCATTTTATTTATATG
8	EMSA/BAS1633 (*yciC*)	CCTTCTCGCCTCCATTTTATTTATATG	GAAATTATTTCTCTCCTTTAAATCG
9	EMSA/BAS1786	CGAGTTATTTCCGTATATTAC	AGCGTAATAATTACGATTTA
10	EMSA/BAS4240 (*rpmG*)	TACAAGGTATAGATTACTTAAATAAAAGG	ATGTAATTCTCCTTTCTTTTAAAG
11	EMSA/BAS1889 (*znuA*)	TCCCCCTCTACACCCTCCTTTC	AAGTTCTCCTCTTTTCTTTTTTTACGG
12	EMSA/*ba zur*	AAAATTATCCAGTAAAAGCC	CGAAATGACAATCCTTC
13	Two-step RT-PCR/BAS4183-BAS4182 (FP83-RP82)	GAATGGAACAAAGTTGGTTATG	CAATACCCATTCCTGCTG
14	Two-step RT-PCR/BAS4182-BAS4181 (FP82-RP81)	GTCACGTGACATTATCGGG	GCTAAGACCTGGATAATCATCC

### Expression and Purification of BaZur

The ORF corresponding to *ba zur* was amplified using *B. anthracis* genomic DNA and gene-specific primers (Table 1) and cloned in the pET28a+ expression vector (Novagen, United States) with a C-terminal 6X-His tag. *E. coli* BL21 (λDE3) cells harboring pET28a-*zur* construct were grown up to early log phase (O.D._600 nm_ ∼ 0.4), induced with 1 mM IPTG at 37°C, and harvested 4 h post-induction. Next, the cells were re-suspended in buffer A (50 mM Tris HCl [pH 8.0], 150 mM NaCl, 5% glycerol) containing 1 mM benzamidine and lyzed by sonication on a Sonics Vibra Cell^TM^ Digital Sonicator. BaZur was purified from the soluble fraction by Ni^+2^-NTA affinity chromatography as described by QIAGEN. The recombinant BaZur was eluted using buffer A containing 300 mM imidazole as 500 μL fractions. The fractions (>95% homogeneity) containing the recombinant protein were pooled and dialyzed in buffer A. The identity of the recombinant protein was substantiated by matrix-assisted laser desorption-time-of-flight (MALDI-TOF) analysis of the trypsin-digested products. The polyclonal sera against BaZur was raised in Swiss-albino mice and the endpoint titer was determined by ELISA. All the mice experiments were done in accordance with the guidelines and with the approval of Jawaharlal Nehru University Institutional Animal Ethical Committee (JNU-IAEC).

Next, the dimeric nature of BaZur was ascertained by SEC. Briefly, the buffer A dialyzed BaZur fractions were concentrated on 3 K MWCO Macrosep advance centrifugal devices (Pall Life Sciences), to a final concentration of 10 mg mL^−1^ and applied to a Superdex-75 pg column (16/600 HR; Amersham Pharmacia Biotech) pre-equilibrated with buffer A, using an automated AKTA FPLC system (Amersham Pharmacia Biotech). The flow rate was maintained at 0.5 mL min^−1^ and the detection wavelength was set at 280 nm. Standard curves were acquired using the gel filtration marker kit (Sigma-Aldrich) in the same buffers and conditions as those in the experimental sample. Concisely, a mixture containing 3 mg each of albumin (MW 66,000 Da), carbonic anhydrase (MW 29,000 Da), and cytochrome C (MW 12,000 Da) in 1 mL of buffer A was used for the standard run. Peaks obtained in the SEC micrographs were integrated and analyzed based upon protein Rt. The molecular weight of the protein fractions obtained from the SEC was extrapolated using appropriate standards by Blue Native-PAGE (BN-PAGE) and SDS-PAGE.

For BN-PAGE, the protein fractions obtained from SEC were mixed with the sample loading buffer (100 mM Tris-HCl [pH 8.0], 50% glycerol, and 0.5% CBB G-250) and incubated at 4°C for 30 min. The mixture was then applied to a pre-casted 15% polyacrylamide gradient gel containing 200 mM Tris-HCl [pH 8.8] and 10% glycerol. For the electrophoretic run, 100 mM histidine containing 0.002% CBB G-250 (pH adjusted to 8.0 using Tris base) and 100 mM Tris-HCl [pH 8.8] were used as the cathode and anode buffers, respectively. BSA, ovalbumin (OVA), and Soybean trypsin inhibitor (STI) were used as native-PAGE standards. The gels were run at 4°C and 25 V/cm with the addition of fresh cathode buffer without CBB G-250 after half of the run. Bands were visualized by Coomassie Brilliant Blue (CBB) staining.

### Real-Time PCR (qRT-PCR)

The growth phase-specific difference in the expression of *ba zur*, the transcriptional profile of *ba zur*, and three regulon genes, namely, *rpmG*, *znuA*, and *yciC* under conditions of zinc excess and TPEN induced zinc deprivation was ascertained by qRT-PCR. Total RNA was isolated from each sample and subjected to DNase treatment as described above. For qRT-PCR, the reaction was put in a total volume of 10 μL, containing 5 μL 2X SYBR green master mix (Life Technologies), 1 μL of 1/10 diluted cDNA (≈5 ng), and 30 nM gene-specific primers (Table 1) designed using Primer Express software, version 2.0 (Applied Biosystems). The PCRs were run in an ABI 7500 using the following program: 50°C for 2 min, 95°C for 10 min, and 40 cycles of 95°C for 15 s, and 60°C for 1 min. Thermal dissociation curves were analyzed in order to detect any non-specific amplification. Since DNA gyrase displays a constitutive expression at all the tested conditions, it was used as an endogenous control for data normalization. The control sample served as the calibrator. The fold change (RQ) in the expression of *ba zur*, *rpmG*, *znuA*, and *yciC* was calculated by the 2^−ΔΔCt^ method. Five experimental repeats were performed and the data plotted represent the mean of the relative quantification obtained from the five runs with SEM.

### *In silico* Regulon Prediction

The homology between BaZur and *B. subtilis* Zur (BsuZur) was determined by BLASTP. Owing to the high identity and similarity between the two, we could convincingly hypothesize that the two might bind to a similar DNA sequence. The 9-1-9 DNA binding consensus sequence, the Zur-box, of BsuZur was used to mine the intergenic regions of the *B. anthracis* genome using the DNA pattern search tool available at: http://embnet.ccg.unam.mx/rsat/dna-pattern_form.cgi by allowing 0/1 mismatch (Nguyen et al., 2018). However, RSAT allows a maximum of one mismatch only, thereby limiting the search. Thus, to make our search even more comprehensive, BLAST analysis was used to determine the homologs of all those important genes which were part of the regulon of BsuZur and Zur of other Gram-positive and Gram-negative bacteria but not obtained in our RSAT search, followed by manual inspection of these genes for the presence of a Zur box with greater than two mismatches. Previously, Gabriel et al. (2008) demonstrated that symmetric mutations at any position from 2–9 resulted in the reduction of the Zur binding. Thus, under no circumstances did we approved symmetric mutations in the two half repeats. Functional annotation of the regulon genes, if required, was done by mapping their domain architecture using the NCBI CDD search (Marchler-Bauer et al., 2014) and UniProt (Consortium, 2016). The promoter prediction of the regulon genes was done using PromBase (Rangannan and Bansal, 2011) and Bprom Softberry^1^. Next, in order to annotate BAS1889 as ZnuA conclusively, the protein sequence was aligned with ZnuA homologs from other bacteria using ClustalW multiple sequence alignment server (Thompson et al., 2003). The presence of transmembrane helices, if any, in the regulon candidate YciC was ascertained by using the Hopp and Woods hydrophilicity plot available at the BioEdit 7.2.1 sequence alignment editor (Hopp, 1989; Hall et al., 2011) and TMHMM software (Krogh et al., 2001).

### Sequence Logo Creation

The Zur box sequence logo for *B. anthracis* was created by multiple sequence alignment of the consensus binding sequence of the six members of the putative BaZur regulon by Clustal Omega (Sievers et al., 2011), followed by entering this alignment as an input into the WebLogo program available at: https://weblogo.berkeley.edu/logo.cgi (Crooks et al., 2004).

### Electrophoretic Mobility Shift Assay (EMSA)

For EMSA, the recombinant BaZur was dialyzed against 20 mM Tris HCl [pH 8.0], 50 mM NaCl, and 5% glycerol. EMSA was performed as described previously (Gopalani et al., 2016) with modifications. Briefly, the probes were prepared by amplifying the upstream region of the respective genes of the predicted regulon using specific primers (Table 1), followed by purifying the amplified fragments, labeling them using T4 polynucleotide kinase and [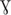-^32^P] ATP (3500 Ci/mmol) and removing the unincorporated nucleotides; 1–1.5 pmoles of each labeled probe was added to the protein in a reaction volume of 50 μL binding buffer (10 mM Tris HCl [pH 7.5], 60 mM NaCl, 20 mM DTT, 10% glycerol, 100 μM ZnCl_2_, 0.5 μg poly (dI-dC)), followed by incubation at 25°C for 20 min. The specificity of the BaZur–DNA interaction was tested by specific and non-specific competition assays. Briefly, for all competition experiments, 50- to 200-fold molar excess of unlabeled specific or non-specific DNA (50X, 100X, and 200X) was added to the binding buffer prior to the addition of radiolabeled probe. NZB DNA lacking the Zur box was used as the negative control. The free probes and the protein-DNA complexes (Zur-DNA complexes) were resolved on a 6% Native-PAGE (29:1 acrylamide: bisacrylamide ratio) at a voltage of 150 V in 0.5X TBE at 4°C, followed by vacuum drying the gels at 70°C for 90–120 min, and visualization and analysis of the radiolabeled probes and Zur-DNA complexes on a Typhoon FLA 9500 phosphorimager.

## Results and Discussion

### Bioinformatics-Based Identification of the *zur* Gene of *B. anthracis* and Mapping Residues Critical for Zinc and DNA Binding

**FIGURE 1:**
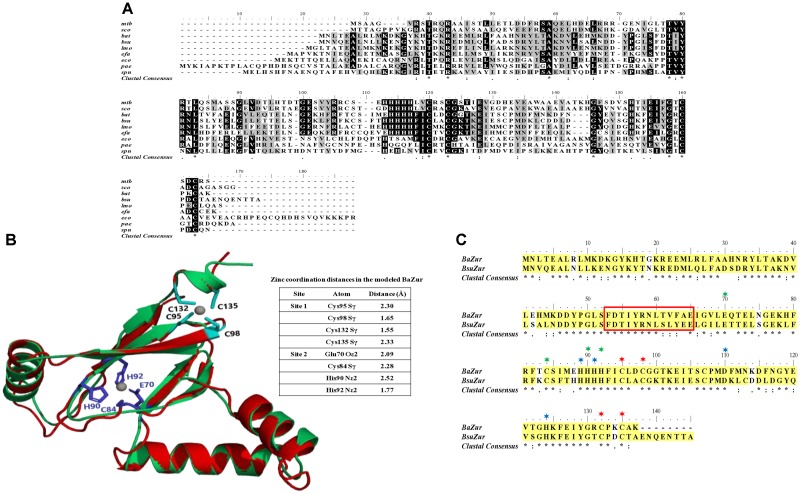
Sequence and structure analysis. **(A)** Multiple sequence alignment. Zur from *B. anthracis* (BaZur) was aligned with Zur archetypes from different bacterial species using CLUSTALW. While the identical residues are marked in black, the similar ones are marked in gray. (^∗^), (:), and (.) indicate the identical residues, conserved mutations, and semi-conserved mutations, respectively. Zur homologs from the following organisms are aligned – *mtb*: *Mycobacterium tuberculosis*; *sco*: *Streptomyces coelicolor*; *bat*: *Bacillus anthracis*; *bsu*: *Bacillus subtilis*; *lmo*: *Listeria monocytogenes*; *efa*: *Enterococcus faecalis*; *eco*: *Escherichia coli*; *pae: Pseudomonas aeruginosa; spn: Streptococcus pneumoniae*. **(B)** Cartoon rendering of the homology model of BaZur monomer (green) superimposed on the template, Zur from *S. coelicolor* (ScoZur) (red). Two Zn^+2^ ions depicted as gray balls bind to each BaZur monomer. The residues constituting the zinc binding site 1 (black) and site 2 (violet) are mapped on the model. **(C)** Pair-wise sequence alignment of BaZur with BsuZur. The two protein sequences were aligned using CLUSTALW and the critical residues comprising zinc binding sites 1 (^∗^), 2 (^∗^), and 3 (^∗^) delineated in BsuZur were mapped onto BaZur. The residues within the red box constitute the DNA binding helix–turn–helix motif. All the alignments were formatted using the Bioedit software available at: http://en.bio-soft.net/format/BioEdit.html.

BAS4181 annotated as the zinc-specific transcriptional regulator was identified in the *B. anthracis* Sterne strain genome by NCBI and KEGG (Kanehisa et al., 2017) databases. The domain architecture reveals that it belongs to the Ferric uptake regulator (Fur) family of proteins with a HTH motif at its N-terminal, implicating it as a sequence-specific DNA binding transcription factor. *B. anthracis* possess three Fur homologs in its genome, namely BAS4001 (Fur), BAS0505 (Per), and BAS4181 (Zur). As observed by BLASTP analysis, BAS4181 holds a similarity of 45 and 52% with Fur and Per, respectively (Supplementary Table S1). This considerable sequence similarity between the fur homologs necessitated additional bioinformatics analysis to authenticate the annotation of BAS4181 as Zur. For this, we aligned the BAS4181 protein sequence with the Zur prototypes from other Gram-positive and Gram-negative bacteria. The multiple sequence alignment depicted high sequence conservation (average similarity of 59%) between BAS4181 and the Zur homologs (Figure 1A). BAS4181 protein sequence exhibited a minimum similarity of 44% and a maximum of 80% with *E. coli* and *B. subtilis* Zur proteins, respectively (Supplementary Table S2).

It has been previously proposed that the zinc binding sites of Zur are distinct from that of the Per and Fur proteins (Traoré et al., 2006). Therefore, despite BaZur exhibiting significant overall sequence similarity with Per and Fur, we selected the fourth best hit, the crystal structure of Zur from *S. coelicolor* (ScoZur) (PDB ID: 3MWM) (Shin et al., 2011) obtained in the BLASTP search at PDB as the template for homology modeling^2^ (Berman et al., 2003) (Supplementary Figure S1). The two proteins had a similarity and an E-value of 50% and 2 × 10^−10^, respectively. The BaZur model superimposed on the template with an RMSD of 0.76 Å over 101 Cα atoms (Figure 1B). In an earlier study, Ma et al. (2011) modeled BsuZur using ScoZur as the template and deduced the zinc binding sites. They demonstrated that purified Zur binds to a maximum of two Zn^+2^ ions per monomer at site 1 and site 2. The site 1 constituted by Cys95, Cys98, Cys132, and Cys135 was shown to be structurally important and any mutation in this site led to the partial or full loss of the repressor activity of the BsuZur. Site 2 comprising of Glu70, Cys84, His90, and His92 was annotated as the sensing site and mutations in this site cause a significant decrease in the zinc binding affinity (Ma et al., 2011). Since BaZur exhibits an overall similarity of 80% with BsuZur, we could extrapolate and functionally annotate zinc binding sites in the BaZur by comparing it with BsuZur. Both site 1 and site 2 constituted by Cys95, Cys98, Cys132, Cys135 and Glu70, Cys84, His90, His92, respectively, could be mapped onto BaZur as well (Figure 1C). The distances from the coordinated residues are also shown (Figure 1B).

Next, in order to get an insight into the BaZur–DNA interaction and delineate the putative residues involved, the BaZur–DNA complex was modeled, with the recognition helix of the HTH occupying the major groove of the DNA (Supplementary Figure S2A). One residue of the turn, i.e., Leu51, and seven residues from the recognition helix of the HTH motif of BaZur, namely, Phe53, Asp54, Tyr57, Arg58, Asn59, Thr61, and Phe63, made close contacts with the DNA residues T3–T9 as well as T28–T31 (Supplementary Figure S2B). The long positively charged side chain of Arg58 in the recognition helix was seen making the largest number of contacts and hydrogen bonds with the base of T31.

Thus, after mapping the critical DNA and zinc binding residues idiosyncratic of Zur proteins on BAS4181, we hereby annotate BAS4181 as *B. anthracis zur* (*ba zur*) and the corresponding protein sequence as BaZur.

### *ba zur* Is Expressed as a Part of a Three-Gene Operon, Which Also Encodes the Components of the ZnuABC System

**FIGURE 2:**
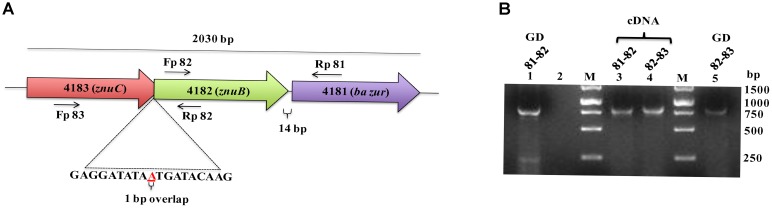
Operonic organization of ba zur. **(A)** Genomic organization of the *ba zur* operon. *ba zur* is part of a three-gene operon. The first gene of the operon is a *znuC* homolog, followed by a *znuB* homolog and lastly *zur*. The translation stop codon of *znuC* overlaps with the start codon of *znuB* (depicted in red). *znuB* and *ba zur* are separated by 14 nucleotides. The black arrows indicate where the RT-PCR primers anneal. **(B)** Reverse transcriptase PCR to detect transcripts from *znuC*-*znuB*-*ba zur* operon. Lanes 3 and 4 represent reverse transcriptase PCR products from *B. anthracis* cDNA. PCR products from *B. anthracis* genomic DNA is shown in Lanes 1 and 5. No product was obtained when RNA without reverse transcription was used as the template (negative control-Lane 2).

The operonic organization of BAS4181 was predicted by DOOR (Mao et al., 2013) and PromBase (Rangannan and Bansal, 2011) databases. While a promoter was predicted upstream of BAS4183, a hairpin loop could be located downstream of BAS4181 with a calculated free energy of formation of −16.3 kcal, suggesting that there is no transcriptional coupling with the gene downstream of *ba zur*. BAS4183, BAS4182, and BAS4181 encode 256, 277, and 137 aa proteins, respectively. BAS4183 when used as a query in BLASTP screen against the NCBI non-redundant (nr) database revealed high similarities with ATP-binding protein of metal transporters, more precisely ZnuC. ZnuC is the ATP-binding protein of a high-affinity zinc ABC transporter, ZnuABC, responsible for providing energy for the zinc uptake (Patzer and Hantke, 1998; Hantke, 2001). Next, in the BLASTP screen of the BAS4182 sequence against the NCBI nr database, it was observed that it exhibits high similarity with the permease of the metal ABC transporter and belongs to ZnuB superfamily (Supplementary Table S3). ZnuB is the permease of ZnuABC transporter which executes the transportation of zinc across the membrane (Patzer and Hantke, 1998; Hantke, 2001). Annotation of BAS4181 has been discussed above. The *in silico* operonic organization was substantiated by RT-PCR. The three genes are oriented in the same direction on the *B. anthracis* genome and are co-transcribed. In a genome loci ABC, if gene B is cotranscribed with both, gene A and C, it could be conclusively stated that gene A, B, and C are part of a single polycistronic unit. The amplification of the regions encompassing BAS4183-BAS4182 and BAS4182-BAS4181 affirms that the three genes are part of a single transcript. For BAS4183-4182, while the forward primer spans 555 nt of BAS4183, the reverse primer covers 331 nt of BAS4182, thereby making the amplicon 886 nt in size. For BAS4182-4181, the forward and the reverse primer spanned 701 nt of BAS4182 and 156 nt of BAS4181, respectively, which resulted in an amplicon of the size 857 nt (Figures 2A,B). There occurs diversity in the operonic organization of *zur* in different bacteria. In *L. monocytogenes*, *S. aureus*, and *P. aeruginosa, zur* is part of a three-gene operon, with other two genes encoding the ATP-binding and permease proteins of the zinc ABC transporter, respectively (Lindsay and Foster, 2001; Corbett et al., 2011; Ellison et al., 2013). In *M. tuberculosis, zur* forms an operon with yet another transcriptional regulator that has been shown to repress the *zur* operon in a zinc-dependent fashion (Maciąg et al., 2007). However, noticeably *zur* is monocistronic in *B. subtilis* and *Staphylococcus epidermidis* (Lindsay and Foster, 2001), and *S. coelicolor* (Shin et al., 2007) (Supplementary Figure S3).

### The Stable Form of BaZur Is a Dimer

**FIGURE 3:**
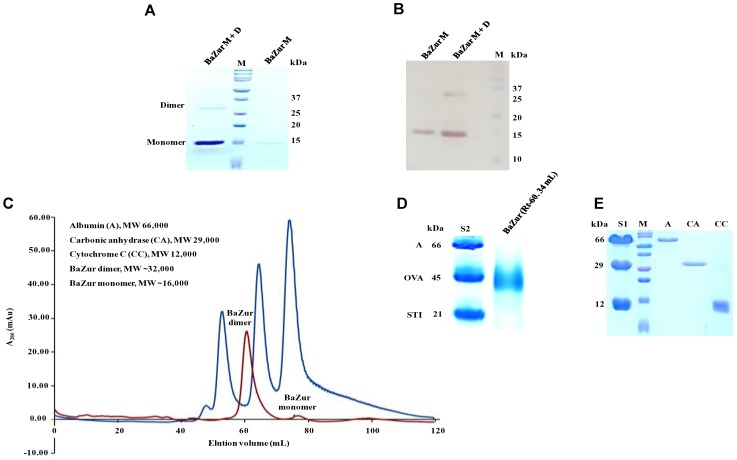
Purification andSEC analysis of BaZur. **(A)** SDS-PAGE analysis of the SEC eluted BaZur, depicting monomeric and dimeric forms. **(B)** Immunoblotting of the purified protein probed with anti-BaZur polyclonal sera. Both monomeric and dimeric forms could be detected. **(C)** SEC micrograph of BaZur (in red) was compared to standard 1 (S1, in blue) containing albumin (A), carbonic anhydrase (CA), and cytochrome C (CC), obtained by fast protein liquid chromatography on a Superdex-75 pg 16/600 sizing column. **(D)** BN-PAGE analysis. Migration of the SEC eluted recombinant BaZur was assessed and compared to standard 2 (S2) containing albumin (A), ovalbumin (OVA), and Soybean trypsin inhibitor (STI). BaZur predominantly migrates as a dimer. **(E)** SDS-PAGE analysis of the SEC eluted standards. M-Bio-Rad precision plus protein TM standard.

The recombinant BaZur was purified from the soluble fraction of BL21 (λDE3) and analyzed on a 15% SDS-PAGE, where it migrated at its expected molecular weight of 17 kDa (Figure 3A). The dimeric propensity of the recombinant BaZur purified by affinity chromatography was observed on SDS-PAGE and by immunoblotting using anti-BaZur polyclonal sera (Figures 3A,B). This was further substantiated by SEC analysis performed on a Superdex-75 pg 16/600 column using the AKTA FPLC system, followed by SDS and BN-PAGE. The experiments were carried out in the absence of detergents and at a low salt concentration of 150 mM NaCl in buffer A. The sequential order of the proteins that passed through the column was: BSA (66 kDa, Rt 53.48), dimeric BaZur (Rt 60.34), CA (29 kDa, Rt 65.02), CC (12 kDa, Rt 74.76), and monomeric BaZur (17 kDa, Rt 75.22). The SEC analysis suggested that under *in vitro* conditions, BaZur exists predominantly in a dimeric state and only feeble absorbance could be recorded corresponding to the monomeric position (Rt 75.22) (Figure 3C). This corroborated with the migration of BaZur in between OVA (45 kDa) and STI (21 kDa) in the BN-PAGE with no detectable band corresponding to the monomeric BaZur (Figure 3D,E). However, when the BaZur fraction (Rt 60.34) was subjected to SDS-PAGE under reducing conditions, it migrated discernibly as a monomer (17 kDa) with a very faint band corresponding to the dimeric BaZur (34 kDa) (Figure 3A). Conclusively, BaZur, the zinc uptake regulator of *B. anthracis* primarily exists in a dimeric state and exhibits attribute similar to the other proteins under reducing and non-reducing conditions. In the SEC micrograph, an overall low absorbance was observed at 280 nm corresponding to 10 mg of recombinant BaZur protein loaded onto the column (Figure 3C), the plausible reason for which was the lack of tryptophan in the protein (Anthis and Clore, 2013).

### BaZur Binds to a Conserved 9-1-9 Sequence, the Zur Box: Characterization of the DNA Binding Ability of BaZur

**FIGURE 4:**
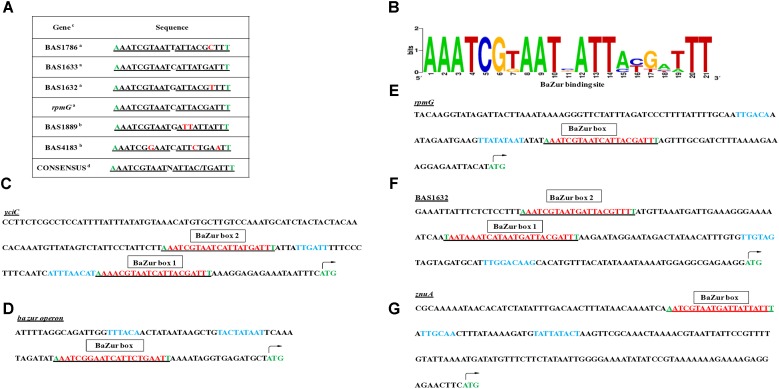
Regulon prediction by computational analysis and manual inspection. **(A)** Table depicting the DNA-binding sites of BaZur (Zur box) in the upstream regions of the putative regulon candidates. a – candidates with 0/1 mismatch in the Zur box, obtained by RSAT. b – candidates with greater than mismatch in the Zur box, identified by manual inspection. Mismatches are denoted in red. c – the Zur box identified for all the listed genes was present in the intergenic region of the *B. anthracis* chromosome. d – consensus was drawn from the binding sites of the putative regulon genes. Note the flanking residues also exhibit conservation and are marked in green. **(B)** Sequence matrix logo. The BaZur DNA binding sites listed in the table were aligned using CLUSTALW, followed by creating the sequence logo using the WebLogo program available at: https://weblogo.berkeley.edu/logo.cgi. **(C–G)** Zur box housed in the upstream regions of the putative regulon candidates: *yciC*, *ba zur* operon, *rpmG*, BAS1632, and *znuA*. Note two Zur boxes (red and underlined) separated by 29 and 33 nucleotides could be located in the upstream region of BAS1632 and *yciC*, respectively. The –10 (proximal to the ATG start codon) and –35 (distal to the ATG start codon) elements of the bioinformatically predicted promoters are shown in blue. The ATG marked in green denotes the translation start codon of the respective gene.

BaZur houses a winged HTH motif and acts as a sequence-specific DNA binding transcription factor. There exists a significant homology between BsuZur and BaZur as indicated by the BLASTP analysis. The two proteins exhibit an identity and similarity of 67 and 80%, respectively. Moreover, out of the 13 residues comprising the HTH motif in both BaZur and BsuZur, nine are identical and three are similar. This prompted us to mine the *B. anthracis* genomic DNA with the Zur box of *B. subtilis* (Gabriel et al., 2008). Using the 0/1 mismatch and manual inspection strategies, we could locate six Zur boxes in the *B. anthracis* genome that regulates 11 genes. Upstream region of BAS1632, BAS1633 (*yciC*), BAS1786, BAS1889 (*znuA*), BAS4183 (*znuB*-*znuC*-*zur* operon), and BAS4240 (*rpmGC*) harbored a minimum of one Zur box (Figure 4A). A sequence logo for the Zur box of *B. anthracis* was derived (Figure 4B). The functional annotation of the candidates of the predicted regulon deciphered their roles in zinc uptake, as metal chaperones, and in zinc mobilization, implicative for maintaining zinc homeostasis in the pathogen. The size of the Zur regulon varies in different bacteria, ranging from nine genes in *Corynebacterium glutamicum* (Schröder et al., 2010) to 17 and 30 genes in *Neisseria meningitidis* (Pawlik et al., 2012), and *M. tuberculosis (*Maciąg et al., 2007), respectively. Exceptionally, Zur regulates up to 154 and 121 genes in *Y. pestis* (Li et al., 2009) and *Streptococcus suis* serotype 2 strain (Feng et al., 2008), respectively. However, these include both the direct as well as the indirect targets of Zur. In the present study, by employing the *in silico* approach, the indirect targets of BaZur, the ones that are regulated despite not being preceded by a Zur box, might have been missed out. However, the indirect targets will be identified by a detailed transcriptomics study involving overexpression of the *ba zur*, currently being conducted by our group. In most of the bacteria, Zur regulon comprises of genes implicated in maintaining zinc homeostasis; however, in *M. tuberculosis*, genes encoding the proteins with immunodominant epitopes like early secretory antigen target 6 (ESAT-6) cluster 3, and ESAT-6/culture filtrate 10 (CFP-10) family are also regulated by Zur (Maciąg et al., 2007). Further, in *S. coelicolor*, Zur regulates a cluster of genes responsible for the synthesis of a siderophore related peptide called coelibactin, deregulation of which inhibits sporulation (Kallifidas et al., 2010). Virulence-related genes like *rovA*, *psaEF*, *psaA*, and *ail* were found to be under Zur regulation in *Y. pestis* (Li et al., 2009).

**FIGURE 5:**
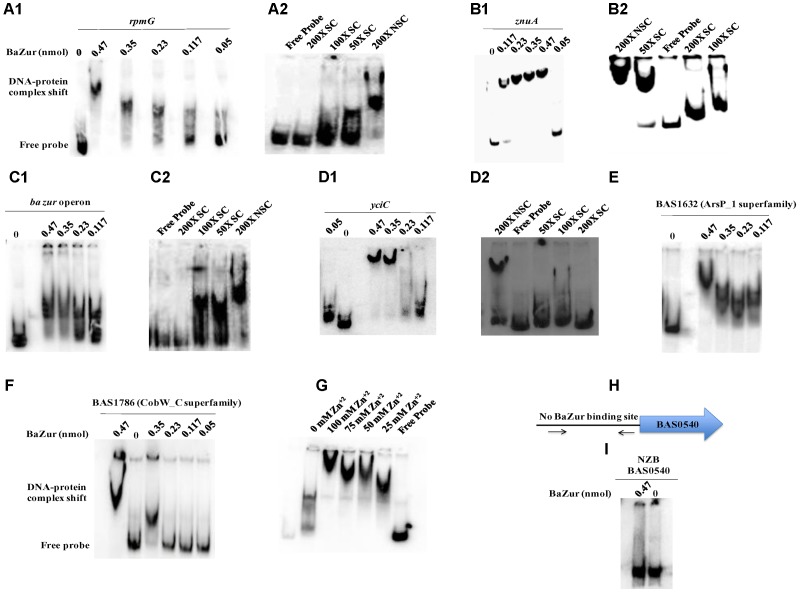
DNA binding ability of BaZur. The DNA binding capability of BaZur was ascertained by EMSA, for which an increasing amount of purified protein was incubated with the upstream regions of the putative regulon candidates housing a Zur box. **(A1,B1,C1,D1,E,F)** Increasing concentration of BaZur (0.05–0.47 nmoles) incubated with the radiolabeled probes (Zur box regions of the regulon genes) of *rpmG*, *znuA*, *znuC*-*znuB*-*ba zur* operon*, yciC*, BAS1632, and BAS1786. **(A2,B2,C2,D2)** Competition with molar excess (50X, 100X, and 200X) of unlabeled specific probe (SC) and with 200X molar excess of unlabeled non-specific probe (NSC). **(G)** Effect of increasing zinc concentration on DNA binding. Increase in the retardation of the target DNA was observed upon increasing the Zn^+2^ from 0 to 100 μM. **(H)** Pictorial representation of the upstream region of the BAS0540-BAS0541 operon that lacks the Zur box. The black arrows indicate the primers used for amplifying this upstream region. **(I)** BaZur incubated with the NZB probe, a 200 bp DNA fragment lacking the Zur box. There was no shift observed even at the highest concentration of BaZur. All the binding reactions were performed at RT and the products were resolved on 6% polyacrylamide native gels in 0.5X TBE at 4°C. The gels were then subjected to vacuum drying at 70°C for 90–120 min. The radiolabeled probes and the protein-DNA complexes were visualized with a storage phosphor screen and analyzed on a Typhoon FLA 9500 phosphorimager. While BaZur could bind to all the test probes, it did not exhibit any binding with the NZB probe, indicative of the specificity of the BaZur–DNA interactions.

Electrophoretic mobility shift assays were performed by incubating increasing concentrations of the recombinant BaZur with the promoter regions of six different genes of the predicted regulon containing the Zur box. The gel mobility shifts enhanced with an increasing protein concentration, such that a complete shift was observed with 6 and 8 μg of BaZur (Figures 5A1,B1,C1,D1,E,F). It can be conjectured that the increasing concentration augments the formation of dimers and higher order polymers resulting in multiple BaZur molecules binding to the Zur box. A gradual increase in the retardation of the target DNA eventuated from incorporating an increasing gradient of Zn^+2^ in the reaction mixtures, the optimum being 100 μM (Figure 5G). This observation corroborates with the previous studies demonstrating the importance of zinc in the DNA binding activity of Zur (Maciąg et al., 2007; Shin et al., 2007; Ellison et al., 2013). However, we could observe the retardation of the target DNA even in the absence of zinc; the plausible reason for which could be the contamination of any divalent cations in the binding reactions or the purified recombinant BaZur already containing some bound Zn^+2^ with it. In the competition experiments, while the addition of the unlabeled specific competitor DNA progressively abolished the shift, there was no effect on the shift upon addition of non-specific unlabeled competitor probe (Figures 5A2,B2,C2,D2). Further, Zur did not exhibit any binding to the NZB probe; a promoter fragment of the BAS0540-BAS0541 operon of *B. anthracis* (Gopalani et al., 2016) which lacks the Zur box (Figures 5H,I). Hence, the binding of BaZur to the promoter of the genes of the predicted regulon was specific.

### Genes Implicated in Zinc Homeostasis Are Regulated by BaZur

Upon manual inspection, a Zur box could be located in the upstream region of BAS1889. Direct binding was observed when purified BaZur was incubated with the upstream region of BAS1889 (Figure 5B). The Zur box was found to be contiguous with the −35 element of the *in silico* predicted promoter (Figure 4G), suggestive of a negative regulation of BAS1889 by BaZur.

BAS1889 is listed as a zinc ABC transporter substrate binding protein (SBP) in the KEGG database. Following stringent bioinformatics analysis, we could annotate BAS1889 as BaZnuA. BAS1889 exhibited 25% identity with *E. coli* ZnuA and an overall average similarity of 46% with other ZnuA homologs (Supplementary Figure S4A). Moreover, the three conserved histidines; H^69^, H^148^, and H^202^ and a histidine-rich stretch, earmark of the ZnuA proteins could be delineated in BAS1889 (Supplementary Figure S4B) (Banerjee et al., 2003; Li and Jogl, 2007; Loisel et al., 2008; Yatsunyk et al., 2008; Ilari et al., 2011). ZnuA, first identified in *E. coli* as an SBP of a high-affinity zinc acquisition system ZnuABC, binds to the Zn^+2^ ions, facilitating its transportation across the membrane (Patzer and Hantke, 1998). The loss of this system and more precisely ZnuA has led to growth defects and attenuation of virulence in many pathogens (Lewis et al., 1999; Campoy et al., 2002; Kim et al., 2004; Yang et al., 2006; Ammendola et al., 2007; Davis et al., 2009; Desrosiers et al., 2010). The conventional arrangement of ABC transporter genes is the presence of a transmembrane permease gene and a gene encoding an ATPase adjacent to the SBP, i.e., the three genes *znuA*, *znuB*, and *znuC* being co-transcribed. However, in *B. anthracis*, the arrangement deviates from the paradigm and while *znuA* is present as a single gene, *znuB* and *znuC* form polycistronic transcripts with *ba zur*. Similar kind of gene arrangement is also seen in *P. aeruginosa*, *Y. pestis*, *Haemophilus influenza*, and *Haemophilus ducreyi*, wherein *znuA* is not co-transcribed with *znuB* and *znuC* (Lewis et al., 1999; Desrosiers et al., 2010; Pederick et al., 2015).

Next, upon manual inspection, we could locate a Zur box downstream of the −35 element of the bioinformatically predicted promoter of the *ba zur-znuB-znuC* operon, hinting toward a negative autoregulation (Figure 4D). We demonstrated a direct binding between BaZur and the promoter region of the operon harboring the Zur box (Figure 5C). Previously, Panina et al. (2003) using *in silico* approaches predicted autorepression of *zur* in *B. anthracis*. However, Zur-mediated autoregulation is rather a non-paradigmatic phenomenon. Like *B. anthracis*, Zur acts as an autorepressor in *E. faecalis*, *P. aeruginosa*, and *L. monocytogenes* (Corbett et al., 2011; Ellison et al., 2013; Latorre et al., 2015). On the contrary, autoregulation has not been observed in *E. coli*, *B. subtilis*, *M. tuberculosis*, and *S. coelicolor* (Maciąg et al., 2007; Shin et al., 2007; Prestel et al., 2015). Thusly, BaZur contemporaneously exerts a negative regulation on all the genes of the high-affinity zinc uptake system ZnuABC of *B. anthracis*. Zur mediated regulation of the high-affinity zinc uptake system ZnuABC or its homologs has also been observed in a number of bacteria like *P. aeruginosa*, *L. monocytogenes*, *E. coli*, *B. subtilis*, and *Y. pestis* (Patzer and Hantke, 1998; Desrosiers et al., 2010; Corbett et al., 2011; Pederick et al., 2015; Prestel et al., 2015).

Further, we could locate a Zur box downstream of the −35 element of the bioinformatically predicted promoter of BAS4240, annotated as *rpmG*, which encodes the L33 ribosomal protein (Figure 4E). Direct binding of BaZur to the upstream region of BAS4240 was shown by EMSA thus indicating toward a negative regulation exerted by BaZur on the transcription of *rpmG* (Figure 5A1). DOOR database indicated BAS4240 as a part of a two-gene operon.

Some of the ribosomal proteins like L31, L33, L36, and S14 are duplicated in a number of bacterial genomes and are often referred to as paralogous proteins that differ from each other in having a zinc-binding motif comprising of two pair of conserved CXXC stretch. While the one harboring this motif has the capability of binding to zinc and is designated as the C^+^ form, the other one lacking this motif is called as the C^−^ form (Makarova et al., 2001). Our *in silico* analysis revealed the existence of three paralogs of *rpmG* in *B. anthracis* genome, namely, BAS4168, BAS0094, and BAS4240 that were identified as homologs of *rpmGA*, *rpmGB*, and *rpmGC*, respectively, based on the sequence similarity with the proteins of *B. subtilis*, *Bacillus licheniformis*, and *Bacillus amyloliquifaciens* (Zeigler et al., 2008; Gabriel and Helmann, 2009). Sequence inspection of all the three members of the *rpmG* family of *B. anthracis* depicted that while RpmGB harbors all the four conserved cysteines, i.e., an intact zinc-binding motif, RpmGC and RpmGA houses only two cysteines, an imperfect motif, incapacitated in zinc binding (Supplementary Figure S5A). Our finding that BaZur exerts a negative regulation upon the C^−^ form of the L33 ribosomal protein of *B. anthracis* based on EMSAs and *in silico* analysis is substantiated by the earlier studies where Zur has been reported to repress C^−^ forms of various ribosomal proteins (Nanamiya et al., 2004; Akanuma et al., 2006; Maciąg et al., 2007; Shin et al., 2007; Gabriel and Helmann, 2009; Li et al., 2009).

In addition to their role in translation, ribosomes also serve as a storehouse for zinc in the cell (Panina et al., 2003; Moore and Helmann, 2005). Bacteria have evolved mechanisms for strategic utilization of this zinc repertoire. Under zinc-depleted conditions, the Zur mediated repression of the genes encoding the C^−^ forms of the ribosomal proteins is released. These C^−^ forms then replace the corresponding C^+^ forms from the ribosomes, resulting in exoneration of zinc, which can then be used by other metalloproteins. This mobilization ensures that even under a zinc famine, the crucial processes and functionality of the proteins that exhibit zinc dependency do not come to a halt (Moore et al., 2005; Akanuma et al., 2006).

Next, we could locate two Zur boxes separated by 33 nucleotides in the upstream region of BAS1633, a homolog of the YciC of *B. subtilis*, of which one almost overlaps the −35 element of the *in silico* predicted promoter while the other one is situated downstream of this element, suggestive of a negative regulation imposed by BaZur on BAS1633 (Figure 4C). Direct binding of BaZur to the upstream region of BAS1633 harboring both the Zur boxes was demonstrated by EMSA (Figure 5D1). Analogously, the upstream region of *bsu yciC* also housed two Zur boxes through which BsuZur could mediate repression. YciC of *B. subtilis* (BsuYciC) is the most reviewed member of the COG0523 subfamily 1. COG0523, divided into 15 subfamilies, belongs to the G3E family of P-loop GTPases, that either function as energy driven insertases or metallochaperones or in some cases as both (Leipe et al., 2002; Haas et al., 2009). Many COG0523 proteins are reported to be regulated by Zur and are implicated in zinc homeostasis (Gaballa and Helmann, 1998; Panina et al., 2003; Haas et al., 2009; Schröder et al., 2010; Napolitano et al., 2012). Pair-wise alignment of BsuYciC and BAS1633 resulted in the delineation of the Walker A, Walker B, and a metal binding CXCC motif in the latter, characteristic of the COG0523 proteins (Supplementary Figure S5B) (Leipe et al., 2002; Haas et al., 2009). Thus, we annotate BAS1633 as YciC of *B*. *anthracis* (BaYciC). YciC homologs have also been identified in *Staphylococcus sp* and *E. faecalis* (Panina et al., 2003).

YciC in *B. subtilis* functions as a zinc chaperone that delivers the metal to YciA, a backup enzyme for folate synthesis utilized by the cell under zinc-depleted conditions. Introduction of *yciC* mutation in a zinc transporter deficient strain worsened the growth defect of the same under zinc-deficient conditions (Gaballa and Helmann, 1998; Lee and Helmann, 2007; Gabriel et al., 2008). In contrast to the membrane dwelling of BsuYciC, BaYciC is a cytoplasmic localized protein. This was indicated by the high hydrophilicity of the protein as determined by the Hopp and woods hydrophilicity analysis (Supplementary Figure S5C). Further, there were no membrane-spanning helices in BaYciC as predicted by TMHMM (Hopp, 1989; Krogh et al., 2001).

Next, we demonstrated direct binding of BaZur to the upstream region of BAS1786 harboring a Zur box downstream of the −35 element of the *in silico* predicted promoter, indicative of a negative regulation (Figure 5F). Domain architecture of BAS1786 as ascertained by SMART database and CDD search available at NCBI indicated it as a COG0523 metallochaperone protein with domains specific to the CobW_C subfamily members. This subfamily 12 derives its name from the protein CobW, the first member that was described and shown to be involved in cobalamin biosynthesis (Crouzet et al., 1991; Blanche et al., 1995; Rodionov et al., 2003; Heldt et al., 2005). However, not all the proteins under this subfamily are true CobW proteins, with many of them being regulated by Zur and implicated in responding to zinc limitation (Haas et al., 2009). Thus, *B. anthracis* has two Zur regulated COG0523 paralogs, analogous to *Pseudomonas putida* (Haas et al., 2009).

Further, we could locate two Zur boxes upstream of the BAS1632 operon, another candidate of the putative BaZur regulon. Direct binding was demonstrated between BaZur and upstream region of BAS1632 housing the two Zur boxes by EMSA (Figure 5E). Both the boxes were positioned upstream of the −35 element of the *in silico* predicted promoter, indicative of a positive regulation by BaZur (Figure 4F). Not much could be inferred from the domain architecture analysis of BAS1632 except that it is a permease of an unknown specificity and belongs to the Arsp_1 superfamily. It exhibits similarity with *Streptococcus mutans* two-component membrane permease complex subunit SMU_747c, involved in low-pH survival, biofilm formation, and acidogenesis.

Hence, we propose a zinc uptake and mobilization model, wherein when *B. anthracis* encounters a zinc famine, the BaZur mediated repression of the genes encoding the ZnuABC system, metallochaperones like YciC and BAS1786 and RpmGC is relieved, actuating their expression. This brings about ferrying of zinc into the cell, intracellular trafficking, shuttling, and mobilization of zinc within the cell, thus delivering it to those zinc-dependent proteins and enzymes that are more crucial for the cellular function. Further, autoregulation of *ba zur* can be envisaged as a mechanism for keeping Zur levels under tight control and stringently maintaining zinc homeostasis in the cell at all the times. Moreover, autoregulation becomes all the more cardinal when *zur* constitutes an operon with the zinc transporter genes.

**FIGURE 6:**
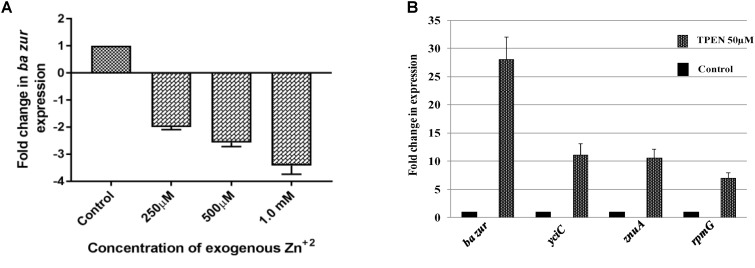
Zinc-dependent transcriptional regulation of *ba zur*, *yciC, znuA*, and *rpmG*. qRT-PCR for quantifying the transcript abundance of **(A)** *ba zur* under conditions of zinc excess, which is expressed as the fold change between treated and control samples. A downregulation in the *ba zur* expression was observed under conditions of zinc excess. **(B)** *ba zur*, *yciC*, *znuA*, *rpmG* under TPEN induced zinc-depleted conditions, which is expressed as the fold change between treated and control samples. An upregulation in the expression was observed. Mean with SEM from five independent runs carried out in triplicates is shown. Statistical significance was determined on the basis of the *p*-value threshold of 0.05, by using the one-way ANOVA followed by multiple comparisons. ^∗∗∗∗^*p* < 0.0001.

Next, in order to add credence to our proposed model, we investigated the expression of some of the crucial regulon genes under TPEN mediated zinc-depleted conditions. While *rpmGC* expression was upregulated by sevenfolds upon induction of zinc-depleted conditions, there was a marked upregulation of 11-folds in the levels of *ba yciC* transcripts and 10-folds in the expression of *znuA* transcripts (Figure 6B). This observation connotes the role of these genes in responding to and combating with the hypo-zincemic conditions.

### Zinc-Dependent Regulation of *ba zur* Operon and Other Regulon Genes

The divaricating expression of *zur* under conditions of zinc depletion in different organisms is intriguing. Hence, we investigated this aspect in *B. anthracis*. For this, cDNA obtained from the *B. anthracis* cells exposed to 50 μM of TPEN, an intracellular zinc chelator, for a period of 40 min was used as a template in qRT-PCR. The *ba zur* expression was upregulated by 28-folds, upon induction of zinc-depleted conditions by TPEN (Figure 6B).

Conversely, we also quantified the *ba zur* transcripts under conditions of zinc excess. For this, cDNA obtained from the cells exposed to an increasing concentration of zinc ranging from 250 μM to 1.5 mM was used as a template in qRT-PCR. At 1.5 mM and higher concentrations of Zn^+2^, the growth was almost inhibited, thereby making RNA isolation an arduous task. The *ba zur* expression decreased by a factor of 2, 2.5, and 3.5 upon exposure to 250 μM, 500 μM, and 1.0 mM, respectively, of exogenous zinc, beyond which the fold change could not be determined (Figure 6A). The downregulation/upregulation was considered significant only when it was >twofolds and the *p*-value was <0.05.

Further, the growth phase-dependent fluctuations in the expression of *ba zur* also corroborated with the above observations. *B. anthracis* growth curve was monitored and four growth points corresponding to the early-exponential (O.D._600 nm_ ∼ 0.3), mid-exponential (O.D._600 nm_ ∼ 0.6), late-exponential (O.D._600 nm_ ∼ 0.9), and the onset of stationary (O.D._600 nm_ ∼ 1.2) phase, respectively, were chosen (Supplementary Figure S6A). There was an inconstancy in the expression of the *ba zur* transcripts as the cells transitioned through the selected growth points, with an increase up to the late-exponential phase, followed by a decrease in the stationary phase (Supplementary Figure S6B). This fluctuation could be attributed to the growth phase-dependent variation in the concentration of zinc in *B. anthracis* cells, which decreases as the cell makes an entry into the late-exponential phase and again begins to rise as the cell enters the stationary phase (Arora et al., 2013). These intracellular zinc-dependent fluctuations in the *ba zur* expression can be envisioned as a mechanism to bring about intracellular zinc homeostasis.

**FIGURE 7:**
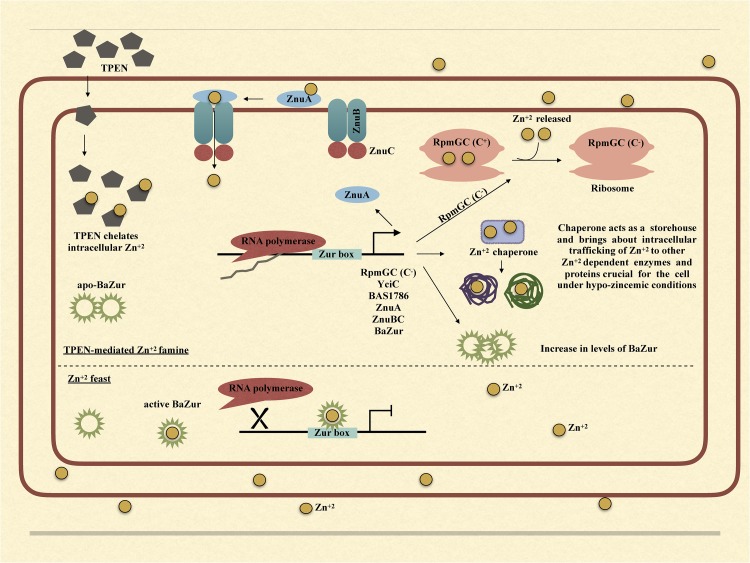
Graphical model illustrating the mechanism of BaZur-dependent regulation of the regulon genes under zinc depleted and zinc excess conditions.

Upon exposure of bacteria to TPEN or exogenous zinc, the expression of *zur* exhibits marked diversity in different organisms. While in some bacteria, it exhibits an upregulation, in others, it either gets downregulated or does not show any change in the expression. There is a zinc-dependent downregulation of *zur* in *E. faecalis* and *P. aeruginosa* (Ellison et al., 2013; Latorre et al., 2015). Antithetically in *S. coelicolor* and *M. tuberculosis*, there occurs a zinc-dependent induction in the *zur* expression (Milano et al., 2004; Canneva et al., 2005; Shin et al., 2007). However, there is no effect on the expression of *zur* under conditions of zinc excess/depletion in *B. subtilis*. The zinc-dependent induction or downregulation of *zur* is either due to the direct autoregulation by Zur itself or because of an indirect regulation exerted by some other regulator. In *E. faecalis* and *P. aeruginosa*, Zur displays a negative autoregulation (Ellison et al., 2013; Latorre et al., 2015). However, in *M. tuberculosis*, the zinc-dependent induction of *zur* is executed by SmtB/ArsR family regulator, which is co-transcribed with *zur* itself (Milano et al., 2004; Canneva et al., 2005; Maciąg et al., 2007). Consonantly, in *S. coelicolor*, there was no Zur-box located in or near the *zur* promoter and thus the zinc-dependent positive regulation was speculated to be brought about by some other unidentified regulator (Shin et al., 2007). In *B. anthracis*, the zinc-dependent downregulation of *zur* can be attributed to the negative autoregulation of the *zur* operon. Thusly, we propose a model; wherein under conditions of zinc excess, BaZur exists in a zinc-bound form, thereby repressing its own expression. This BaZur-mediated repression is released when zinc concentration decreases, which results in the existence of a non-zinc bound form of BaZur (the apo-BaZur) that either possess an attenuated or no DNA binding capability at all (Figure 7). This negative autoregulation of *znuB*-*znuC*-*ba zur* operon by BaZur can be foreseen as an additional control mechanism for tightly regulating the expression of genes implicated in zinc homeostasis in the pathogen.

## Conclusion

Owing to the multifaceted roles that zinc plays in the bacteria, both intracellular and extracellular zinc homeostasis is cardinal for its survival. Zur is a protein indispensable for maintaining zinc homeostasis. Our study provides an insightful investigation of the previously unexplored Zur of *B. anthracis* and its connotation in maintaining zinc homeostasis in the pathogen. Conclusively, we propose a model, wherein, under the conditions of zinc feast, BaZur binds to zinc and represses the expression of its regulon genes. However, under conditions of zinc famine, BaZur predominantly exists in a zinc-free form, the apo-BaZur, which is either incapable or enfeebled for DNA binding. Thus, the BaZur mediated repression is prorogued, resulting in the expression of the genes encoding the components of a high-affinity zinc uptake system, the C^−^ form of the ribosomal protein, and the metal chaperones acting as zinc depot, thereby actuating an adaptive response to the hypo-zincemic conditions (Figure 7). Further, the negative autoregulation exerted by BaZur provides an additional level of control on the Zur mediated trancriptomic rewiring in *B. anthracis*.

## Author Contributions

RB and DK conceptualized the study. RB, DK, and MoG planned and performed all the experiments and analyzed the data. DK and MoG primarily wrote the manuscript with RB and SB providing intellectual inputs. SB performed the homology modeling of BaZur and BaZur–DNA complex. MaG performed the SEC analysis of BaZur. HJ provided active participation in the revision of the manuscript, performing some crucial experiments.

## Conflict of Interest Statement

The authors declare that the research was conducted in the absence of any commercial or financial relationships that could be construed as a potential conflict of interest.

## Footnotes

1 http://www.softberry.com/berry.phtml?topic=bpromandgroup=programsandsubgroup=gfinb

2 www.wwpdb.org

## Abbreviations

aa: amino acids
AP: alkaline phosphatase
*ba zur*: *Bacillus anthracis* zinc uptake regulator gene
BaZur: *Bacillus anthracis* zinc uptake regulator protein
BCIP: 5-bromo-4-chloro-3’-indolyphosphate
BN-PAGE: blue native polyacrylamide gel electrophoresis
ELISA: enzyme linked immunosorbent assay
EMSA: electrophoretic mobility shift assay
HRP: horseradish peroxidase
NBT: nitro-blue tetrazolium
Rt: retention time
SEC: size exclusion chromatography
TPEN, N, N, N′: N′-tetrakis (2-pyridylmethyl) ethylenediamine.
