# Supplementary figures and images for: Identification, Functional Characterization, and Regulon Prediction of the Zinc Uptake Regulator (zur) of Bacillus anthracis – An Insight Into the Zinc Homeostasis of the Pathogen
Source: Front Microbiol. 2019 Jan 11;9:3314. doi: 10.3389/fmicb.2018.03314 (PMC6336718; doi:10.3389/fmicb.2018.03314)

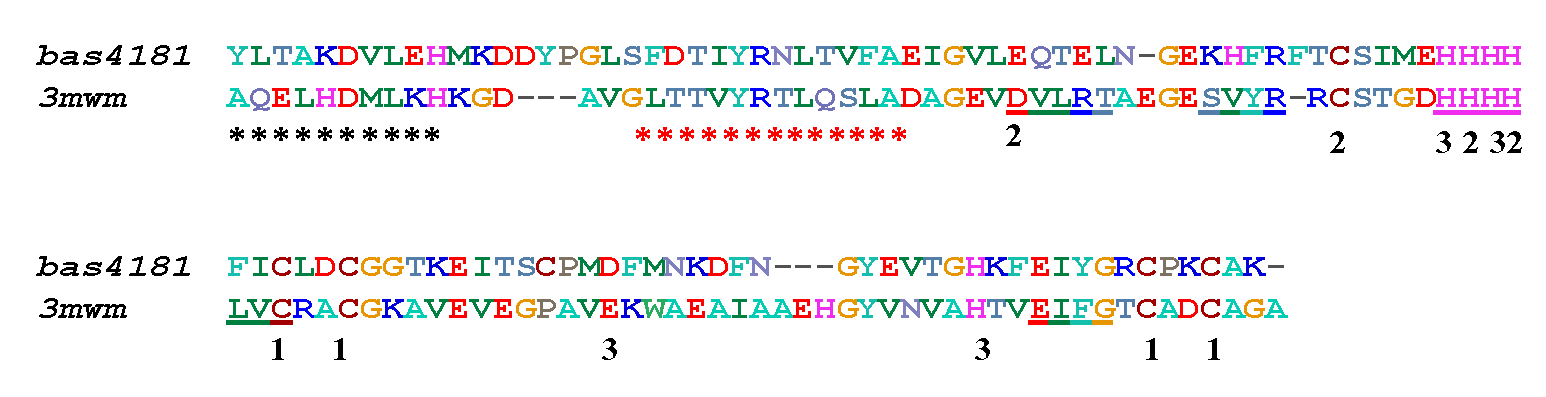

Supplement: Supplementary file 1 [file Image_1.TIF]

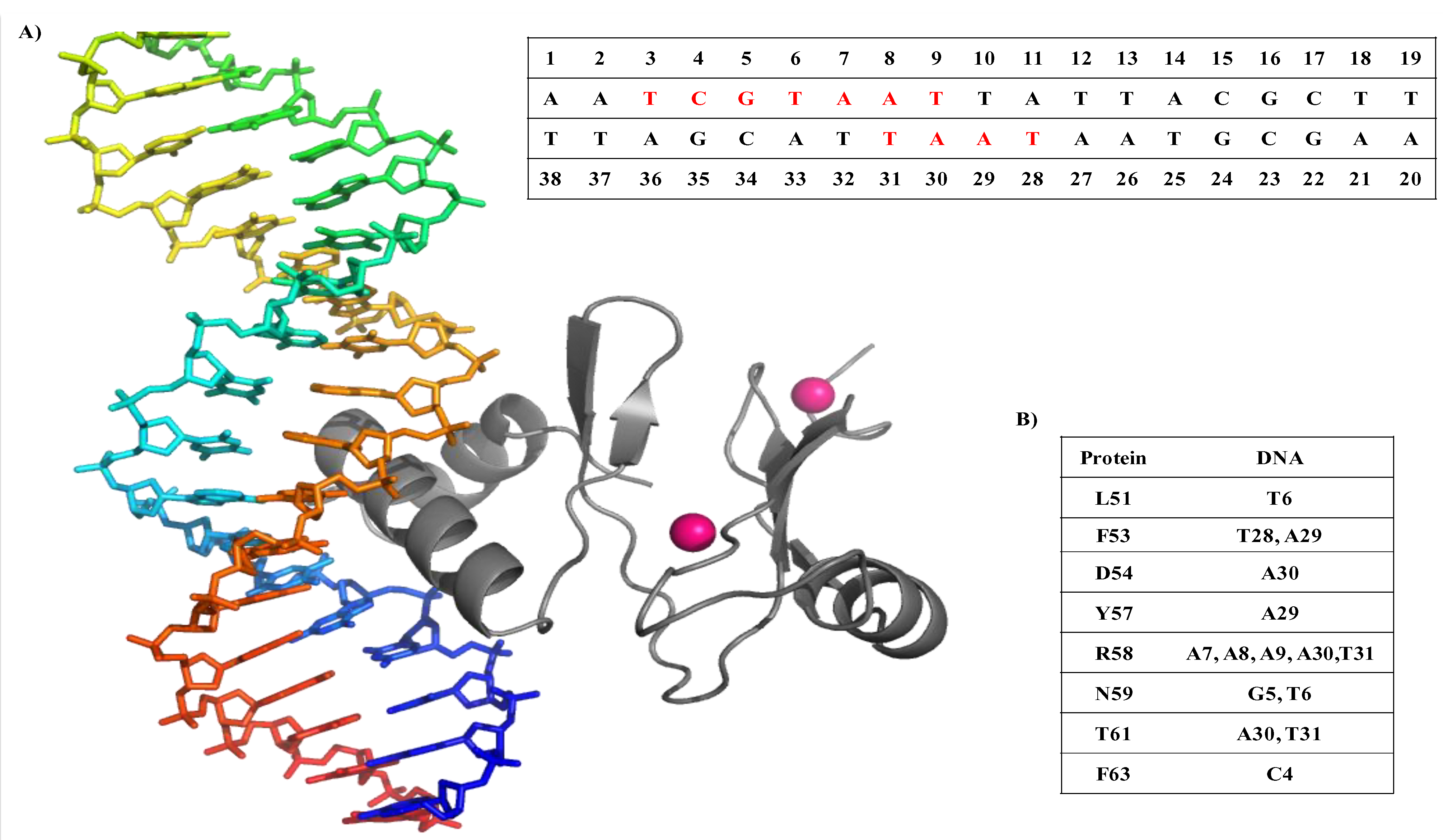

Supplement: Supplementary file 2 [file Image_2.TIF]

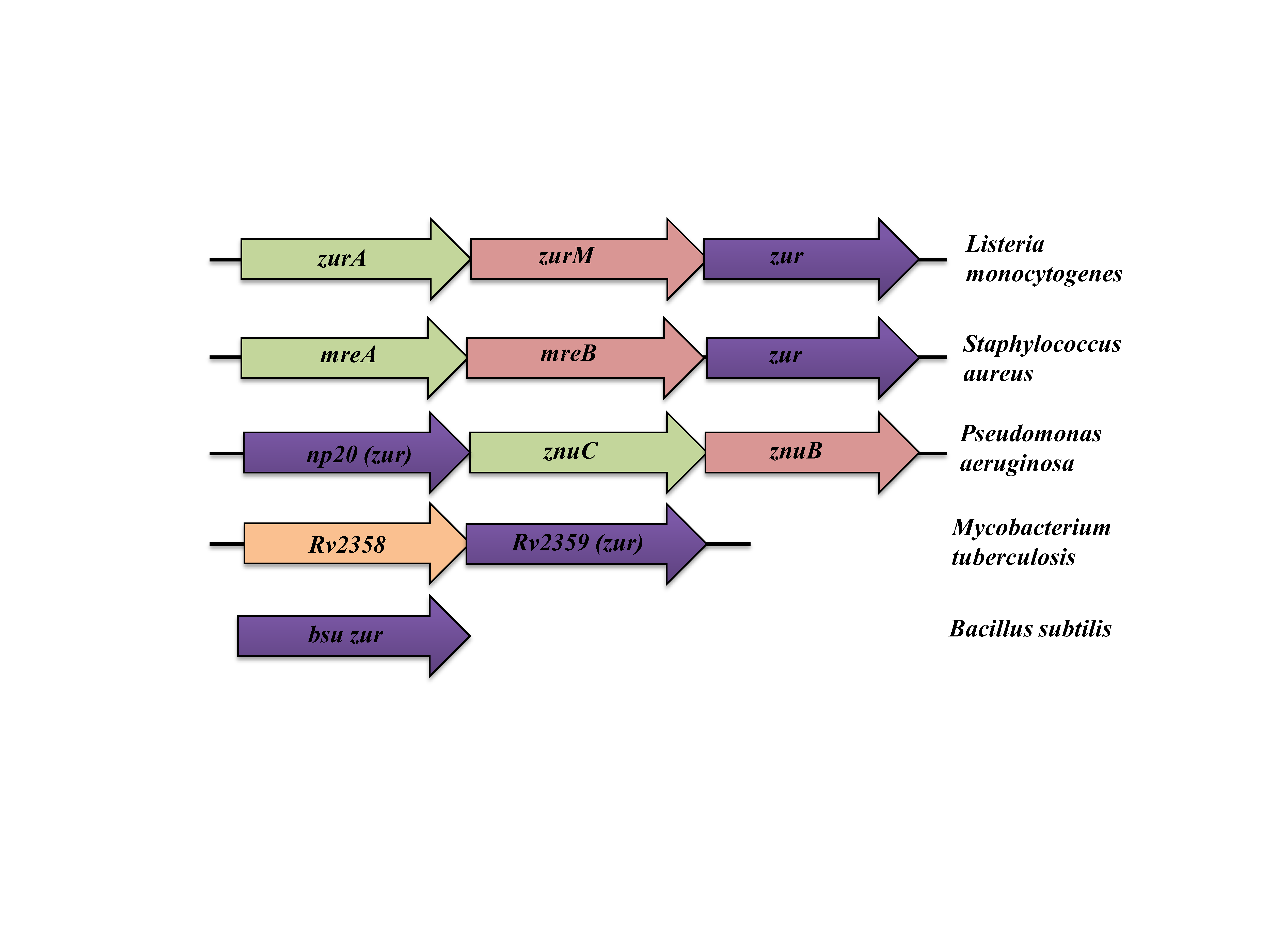

Supplement: Supplementary file 3 [file Image_3.TIF]

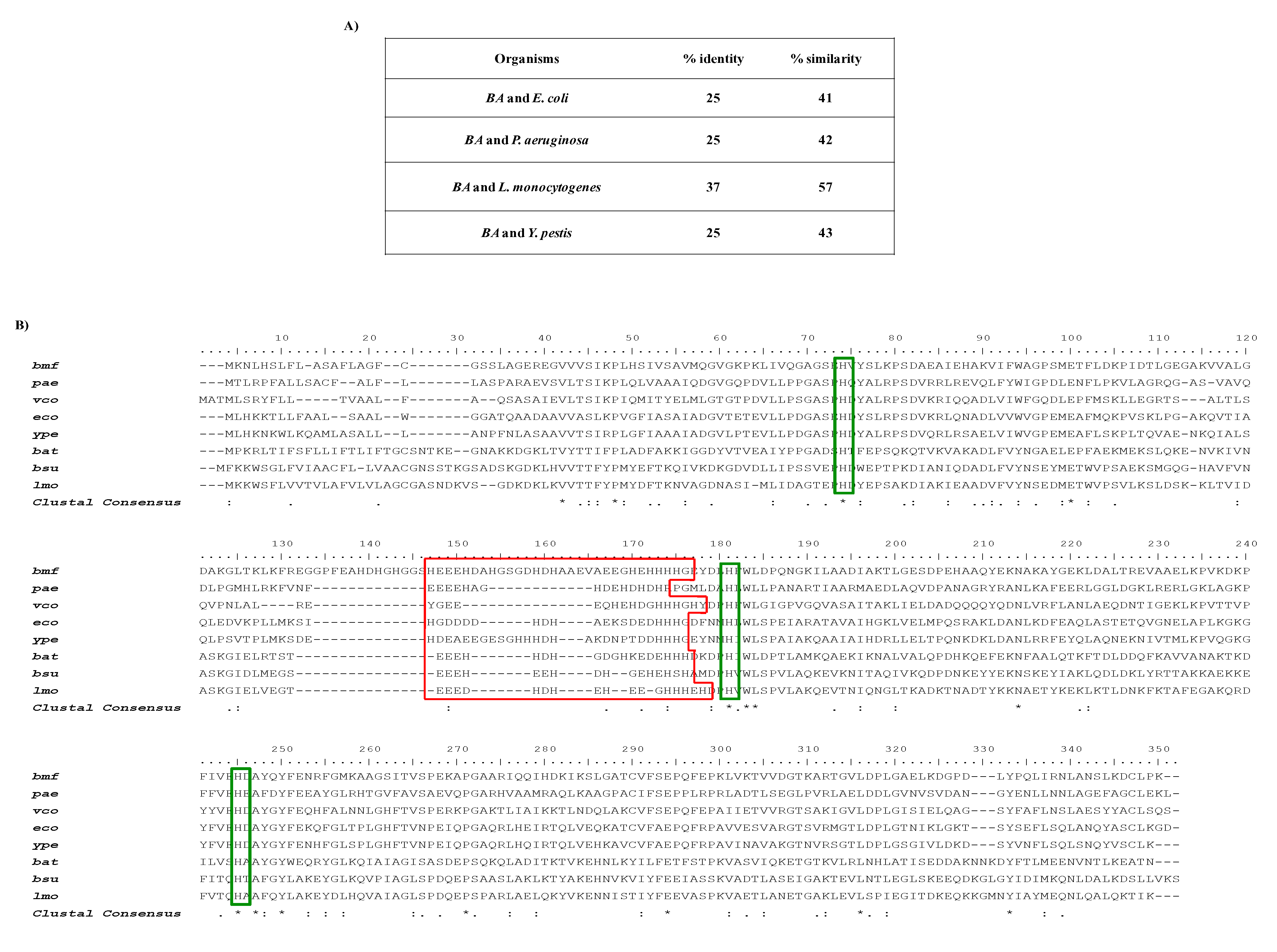

Supplement: Supplementary file 4 [file Image_4.TIF]

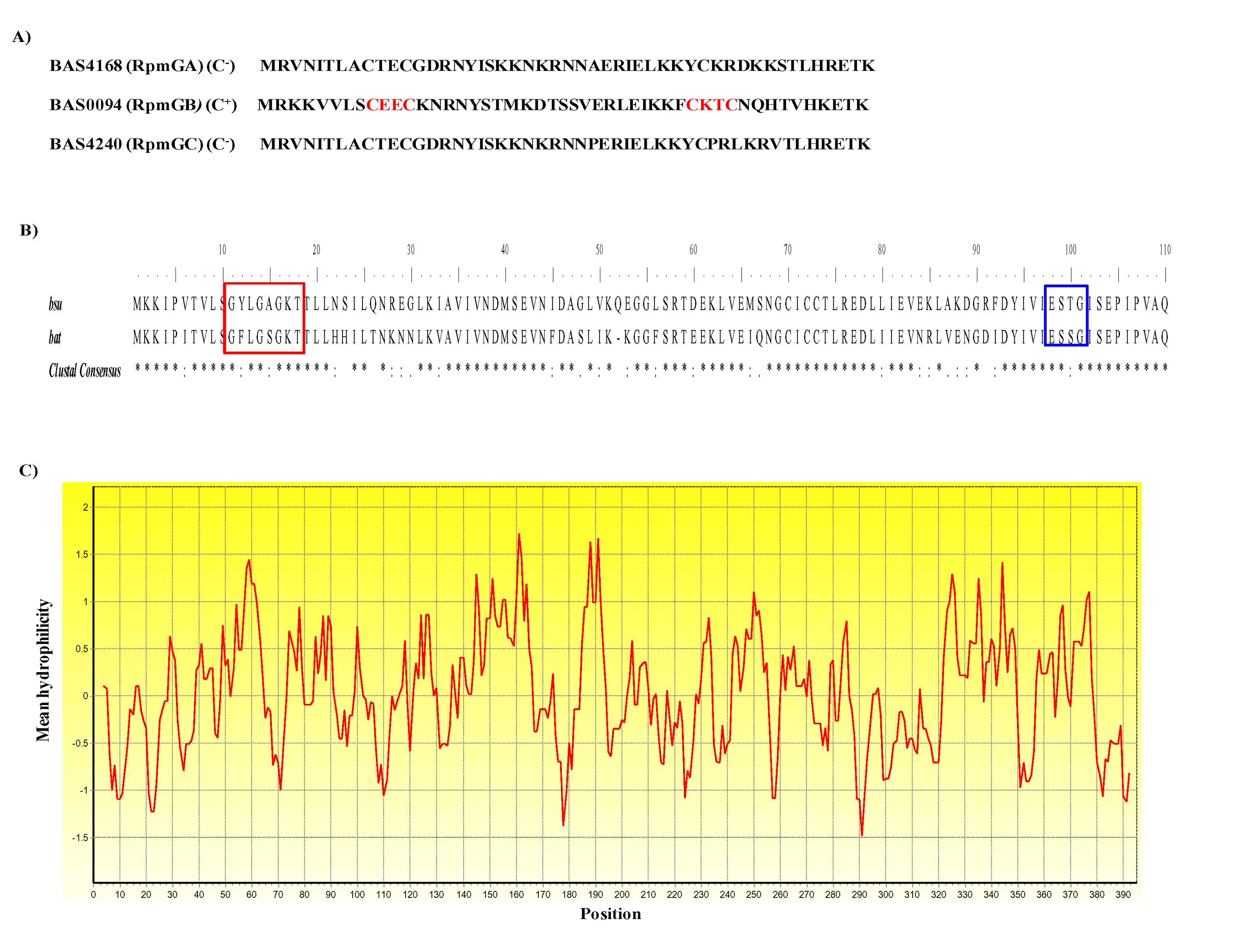

Supplement: Supplementary file 5 [file Image_5.TIF]

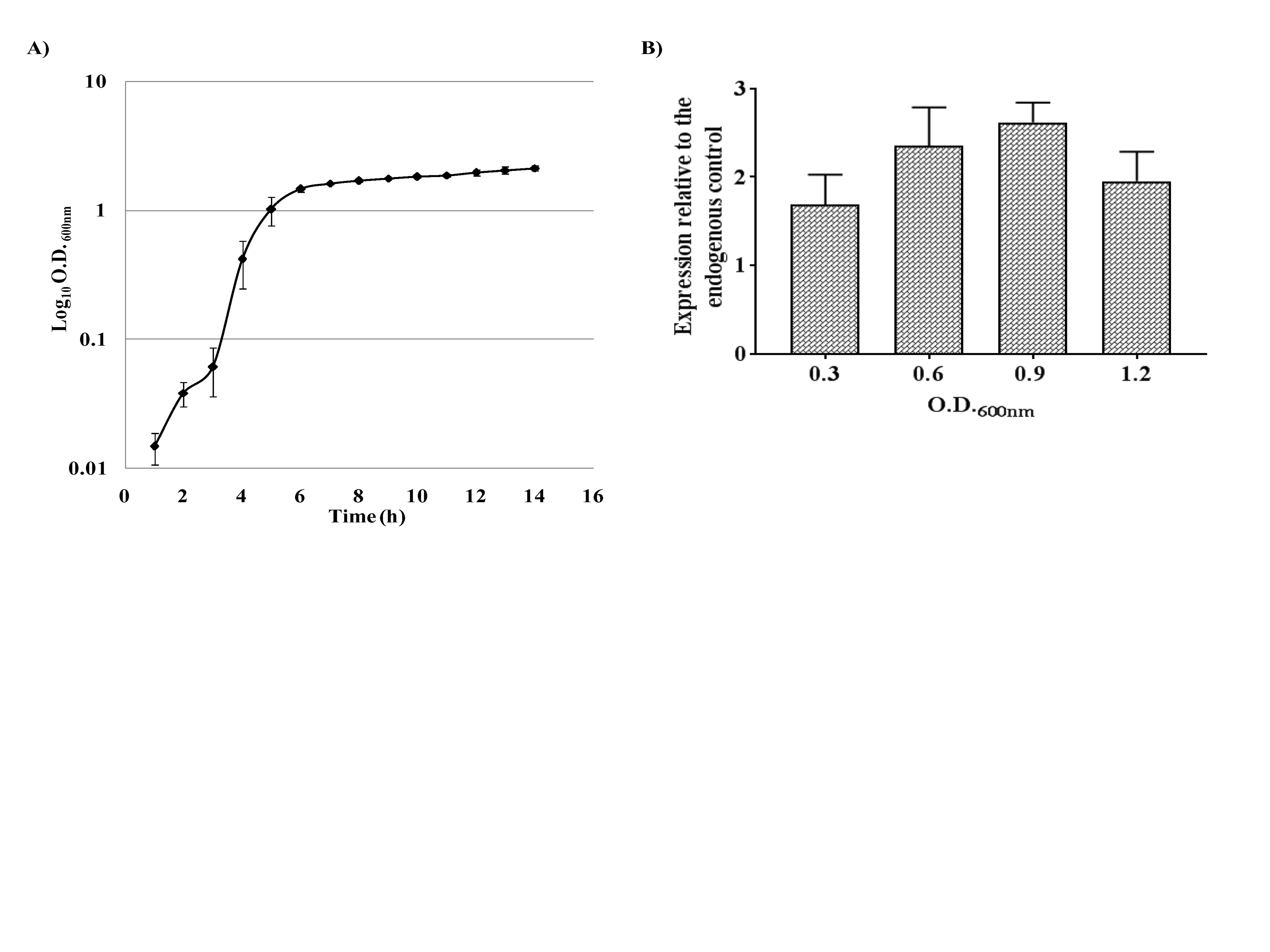

Supplement: Supplementary file 6 [file Image_6.TIF]
