# Supplementary material for: Identification, Functional Characterization, and Regulon Prediction of the Zinc Uptake Regulator (zur) of Bacillus anthracis – An Insight Into the Zinc Homeostasis of the Pathogen
Source: Front Microbiol. 2019 Jan 11;9:3314. doi: 10.3389/fmicb.2018.03314 (PMC6336718; doi:10.3389/fmicb.2018.03314)
Supplement: Supplementary file 7 [file Table_1.DOCX]

**Supplementary Information**

**Supplementary Table 1**: Percentage identity and similarity between BaZur and other Fur family proteins of *B. anthracis*

| **Gene name** | **% identity** | **% similarity** |
| --- | --- | --- |
| **BaZur and BaPerR** | **30** | **52** |
| **BaZur and BaFur** | **27** | **45** |

**Supplementary Table 2**: The table depicts % identity and % similarity between BaZur and its homologs. The E-value for each BLAST analysis is also mentioned.

| **Organisms** | **% identity** | **% similarity** | **E-value** |
| --- | --- | --- | --- |
| ***BA* and *Escherichia coli*** | **23** | **44** | **2e^-11^** |
| ***BA* and *Streptomyces coelicolor*** | **37** | **50** | **9e^-16^** |
| ***BA* and *Bacillus subtilis*** | **67** | **80** | **2e^-72^** |
| ***BA* and *Listeria monocytogenes*** | **61** | **79** | **4e^-68^** |
| ***BA* and *Mycobacterium tuberculosis*** | **25** | **46** | **2e^-15^** |
| ***BA* and *Enterococcus faecalis*** | **46** | **66** | **4e^-47^** |
| ***BA* and *Streptococcus pneumoniae*** | **25** | **47** | **8e^-15^** |

**Supplementary Table 3**: Table depicting BAS4183 and BAS4182 as homologs of ZnuC and ZnuB, respectively. The E-values of the BLASTP analysis and average % identity are also shown.

| **Proteins** | **E-value** | **Average % identity** |
| --- | --- | --- |
| **BAS4183 and ZnuC** | **9.07e^-114^** | **41.1** |
| **BAS4182 and ZnuB** | **8.59e^-63^** | **35.4** |

**Supplementary Figure 1**. Pair-wise sequence alignment of BaZur with ScoZur. The two protein sequences were aligned using CLUSTALW and the critical residues comprising zinc binding sites 1, 2, and 3 have been marked. The alignment was formatted using the Bioedit software available at <http://www.mbio.ncsu.edu/bioedit/bioedit.html>.

**Supplementary Figure 2. Modeling of the BaZur-DNA complex**. **(A)** The BaZur-DNA complex showing the recognition helix of BaZur in the major groove of the consensus sequence (Zur box) housed in the upstream region of BAS1786. The numbering scheme is shown in the inset. While the DNA is shown as a stick, the protein is shown as a grey cartoon with Zn^+2^ as pink balls. The first cluster from the HADDOCK 2.2 results had a score of -121.6 + 5.3 and the largest cluster size of 101. **(B)** Table depicting the residues of the protein and DNA involved in the interaction.

**Supplementary Figure 3. Comparison of the genomic organization of *zur* in different bacteria.** The *zur* loci display considerable diversity in different organisms ranging from being a single gene to a part of two and three-gene operons.

**Supplementary Figure 4.**  **BAS1889 is a ZnuA homolog in *B. anthracis*.** (A) Table depicting the % identity and % similarity of BAS1889 with ZnuA homologs from other bacterial species. (B) Multiple sequence alignment. BAS1889 was aligned with ZnuA prototypes from different bacterial species using CLUSTAL OMEGA. The conserved histidine-rich strech is marked by a red box. The histidine residues within the green boxes are the hallmark of the ZnuA family of proteins. His69, His148, and His212 are the three c=onserved residues of BaZnuA. (*), (:), (.) indicates the identical residues, conserved mutations, and semi-conserved mutations, respectively. ZnuA homologs from the following organisms are aligned- *bmf*: *Brucella abortus*; *pae: Pseudomonas aeruginosa, vco: Vibrio cholera; eco*: *Escherichia coli; ype: Yersinia pestis; bat*: *Bacillus anthracis; bsu*: *Bacillus subtilis; lmo*: *Listeria monocytogenes*.

**Supplementary Figure 5. Analysis of the putative regulon candidates**. **(A)** Sequence analysis of the *rpmG* paralogs of *B. anthracis*. There exist three paralogs of *rpmG* encoding the ribosomal L33 protein in *B. anthracis*. While BAS4240 and BAS4168 are the C^-^ forms, BAS0094 is the C^+^ form. The two forms differ in having the zinc ribbon motif comprising of two pair of conserved CXXC motifs in the protein sequence. **(B)** Pair-wise alignment of YciC of *B. anthracis* (*bat*) and *B. subtilis* (*bsu*). The two sequences were aligned using CLUSTALW. The residues enclosed within the red and blue boxes constitute the Walker A and Walker B motifs, respectively, idiosyncratic of the COG0523 family of proteins. **(C)** Hopp and Woods hydrophilicity plot for the analysis of the degree of hydrophobicity/hydrophilicity of YciC. The sequence predominantly consists of hydrophilic regions as indicated by the positive mean hydrophilicity values on the Y-axis.

**Supplementary Figure 6.**  **(A)** Growth curve of *B. anthracis* monitored in BHI broth. **(B)** qRT-PCR for *ba zur* transcripts from total RNA of *B. anthracis* (O.D._600nm_~ 0.3, 0.6, 0.9, 1.2). Normalized Ct values are plotted against different growth stages. Mean with SEM values, from five independent experiments carried out in triplicates are shown.
